# Supplementary material for: A Newcastle disease virus expressing a stabilized spike protein of SARS-CoV-2 induces protective immune responses
Source: Nat Commun. 2021 Oct 27;12:6197. doi: 10.1038/s41467-021-26499-y (PMC8551302; doi:10.1038/s41467-021-26499-y)
Supplement: Supplementary file 1 — Supplementary Information [file 41467_2021_26499_MOESM1_ESM.pdf]

Supplementary Table 1. Primers used to introduce Hexa Pro mutations into the spike.

Primer name	Primer sequence 5’ to 3’
Hex 1 For	GCACCGAGTTCCCCCCC
Hex 1 Rev	GTCCTCGATAGGGCTCCGCTTGCTGGGC
Hex 2 For	AAGCGGAGCCCTATCGAGGACCTGCTGTTC
Hex 2 Rev	AAAGGGGATCTGCAGAGCAGGGCCAGCTCCA
Hex 3 For	CTGCTCTGCAGATCCCCTTTCCAATGCAGATGGC
Hex 3 Rev	AGGGCGCTGGGTGTGCTGCTCAGG
Hex 4 For	AGCAGCACACCCAGCGCCCTGGGAAAGC
Hex 2P Rev	GGGCCACTTGATGTACTGCTCG
Hex 2P For	AGCAGTACATCAAGTGGCCCG
Hex 5 Rev	GAGAGTTGGACCTTGGGTCCG
Transgene For	CGAGTTCCCCCCCGC GGTTAGAAAAAATACGGGTAGAAGCCGCCACCATGTTCGTGTTTCTGGTGC
Transgene Rev	GAGAGTTGGACCTTGGGT CCGCGG TTATCACATCTTTGTAGTTGC

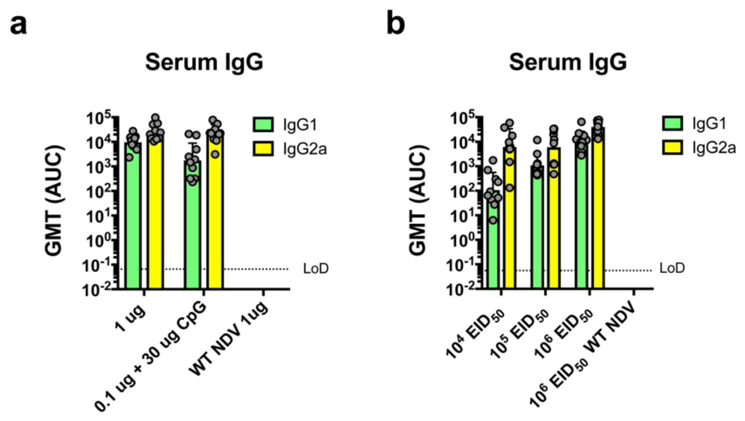

Supplementary Figure 1. Vaccination with inactivated and live NDV-HXP-S favors a IgG2a production over IgG1. ELISAs were performed using either anti-mouse IgG1 (green) or IgG2a (yellow) secondary antibody to determine subclasses of IgG induced by **a** inactivated NDV-HXP-S related to the mouse study (n=10) described in Figure 2 or **b** live NDV-HXP-S related to the mouse study (n=10) described in Figure 6. GMT AUC was plotted. The error bars represent geometric SD.

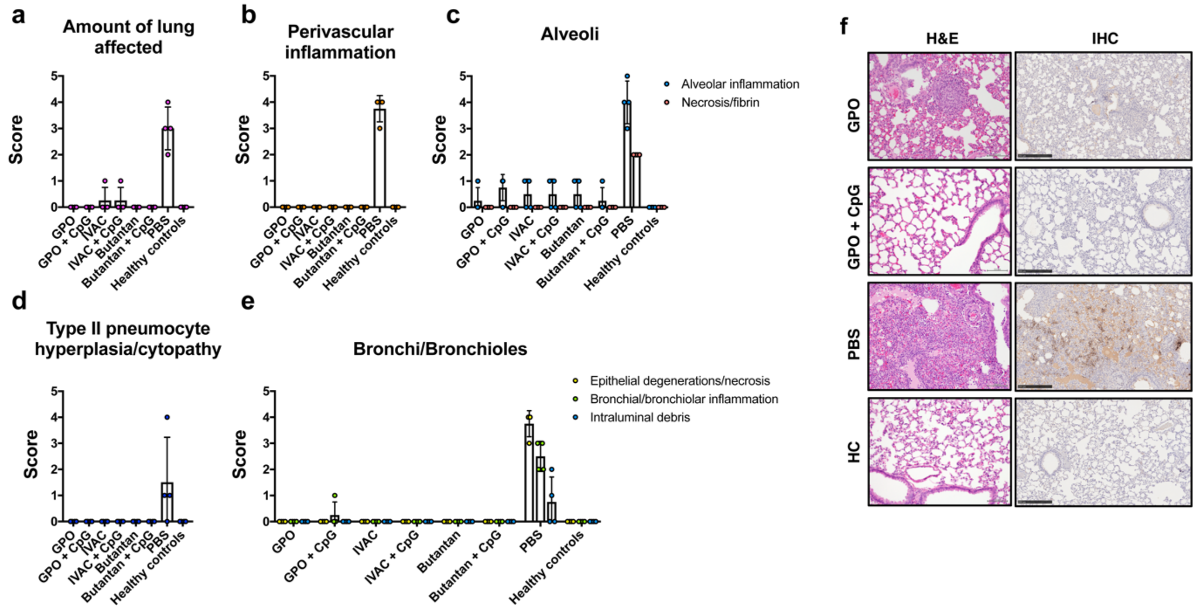

Supplementary Figure 2. Inactivated NDV-HXP-S reduces SARS-CoV-2 induced lung pathology in hamsters. Related to the hamster study in Figure 4, Left lung lobes of hamsters (n=4) collected at day 5 post-challenge were fixed in neutral buffered formalin and cut into 5µm sections and stained with hematoxylin and eosin (H&E). All sections were evaluated by a veterinary pathologist who was blinded to the vaccination groups to score **a** Amount of lung affected; **b** Perivascular inflammation; **c** Alveolar inflammation and necrosis/fibrin; **d** Type II pneumocytes hyperplasia/cytopathy and **e** Epithelial degeneration/necrosis, bronchial/bronchiolar inflammation, intraluminal debris. The error bars represent SD. **f** Examples of H&E staining and IHC staining of lung samples. Lungs samples collected at day 5 post challenge representing the unadjuvanted groups (GPO), the adjuvanted groups (GPO + CpG), the negative control groups (PBS) and the healthy control groups (HC) were shown. The brown color in the IHC indicates the presence of the N protein of SARS-CoV-2. The scale bar represents 100 µM in the H&E (bottom right) images and 250 µM in the IHC (bottom left) images.

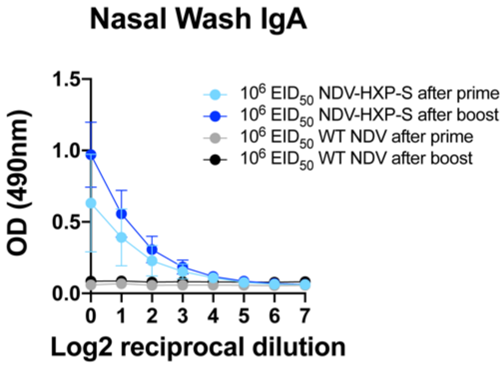

Supplementary Figure 3. Live NDV-HXP-S induces spike-specific mucosal IgA. Nasal washes were collected from mice that were vaccinated with 10^6 EID_{50} of NDV-HXP-S (n=5) or WT NDV (n=4, negative control) at 3 weeks after the intranasal prime (light blue and grey) and 3 weeks after the intramuscular boost (dark blue and black). Spike-specific IgA in the nasal washes was measured by ELISAs. The error bars represent SD.

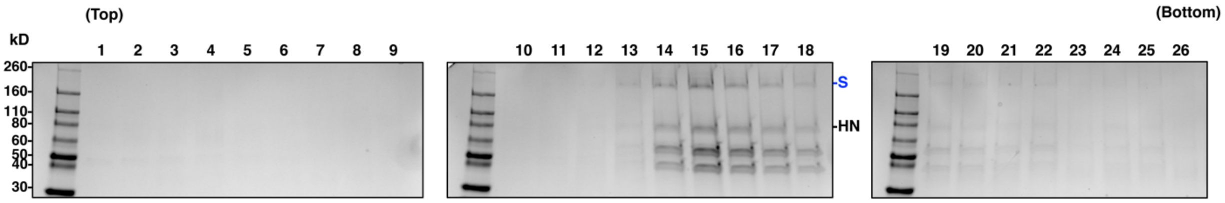

Supplementary Figure 4. The spike protein associates with the NDV particles. A sucrose-gradient (10 - 60%) fractionation was performed via ultracentrifugation. Reducing SDS-PAGE (4-20%) followed by Coomassie Blue staining was performed to examine the presence of spike protein and NDV viral proteins in each fraction. A total of 26 fractions from top to bottom was collected. The spike protein (S, blue) and NDV HN protein was indicated in the gel images. Images from one out of the two independent experiments were shown.

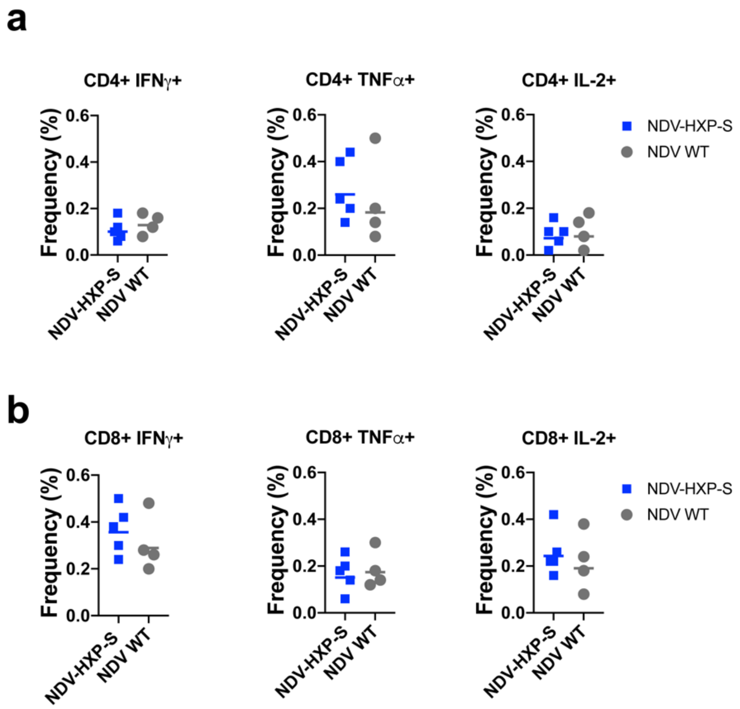

Supplementary Figure 5. Spike-specific T cells responses. Mice were vaccinated with 10^6 EID_{50} of the NDV-HXP-S (n=5, blue) or 10^6 EID_{50} of the WT NDV (n=4, negative control, grey) following an intranasal prime - intramuscular boost regimen. Spleens were collected from mice at 3 weeks after the boost. The splenocytes were stimulated with spike-specific peptides and intracellular cytokine staining was performed. **a** The frequency of CD4+IFNγ+, CD4+TNFα+ and CD4+IL-2+ cells of the total cells and **b** the frequency of CD8+IFNγ+, CD8+TNFα+ and CD8+IL-2+ cells of the total cells were shown.
