# Supplementary material for: A Newcastle disease virus expressing a stabilized spike protein of SARS-CoV-2 induces protective immune responses
Source: Nat Commun. 2021 Oct 27;12:6197. doi: 10.1038/s41467-021-26499-y (PMC8551302; doi:10.1038/s41467-021-26499-y)
Supplement: Supplementary file 3 — Reporting Summary [file 41467_2021_26499_MOESM3_ESM.pdf]

nature portfolio

Corresponding author(s): Peter Palese

Last updated by author(s): Sep 26, 2021

Reporting Summary

Nature Portfolio wishes to improve the reproducibility of the work that we publish. This form provides structure for consistency and transparency in reporting. For further information on Nature Portfolio policies, see our Editorial Policies and the Editorial Policy Checklist.

Statistics

For all statistical analyses, confirm that the following items are present in the figure legend, table legend, main text, or Methods section.

n/a	Confirmed	
☐	☒	The exact sample size (*n*) for each experimental group/condition, given as a discrete number and unit of measurement
☐	☒	A statement on whether measurements were taken from distinct samples or whether the same sample was measured repeatedly
☐	☒	The statistical test(s) used AND whether they are one- or two-sided<br>*Only common tests should be described solely by name; describe more complex techniques in the Methods section.*
☒	☐	A description of all covariates tested
☐	☒	A description of any assumptions or corrections, such as tests of normality and adjustment for multiple comparisons
☐	☒	A full description of the statistical parameters including central tendency (e.g. means) or other basic estimates (e.g. regression coefficient) AND variation (e.g. standard deviation) or associated estimates of uncertainty (e.g. confidence intervals)
☐	☒	For null hypothesis testing, the test statistic (e.g. *F*, *t*, *r*) with confidence intervals, effect sizes, degrees of freedom and *P* value noted<br>*Give P values as exact values whenever suitable.*
☒	☐	For Bayesian analysis, information on the choice of priors and Markov chain Monte Carlo settings
☒	☐	For hierarchical and complex designs, identification of the appropriate level for tests and full reporting of outcomes
☒	☐	Estimates of effect sizes (e.g. Cohen's *d*, Pearson's *r*), indicating how they were calculated

Our web collection on statistics for biologists contains articles on many of the points above.

Software and code

Policy information about availability of computer code

Data collection	The raw data was shown in Microsoft Excel (Version 16.16.27) spreadsheet files. The flow cytometry data was acquired using SpectroFlo software. The SDS-PAGE was imaged using Image Lab Version 6.0.1
Data analysis	The data was analyzed using GraphPad Prism Version 7.0e or Version 8.0.2. The flow cytometry data was analyzed using DeNovo FCS express Research Version 7.06.0015 and GraphPad Prism Version 8.0.2

For manuscripts utilizing custom algorithms or software that are central to the research but not yet described in published literature, software must be made available to editors and reviewers. We strongly encourage code deposition in a community repository (e.g. GitHub). See the Nature Portfolio guidelines for submitting code & software for further information.

Data

Policy information about availability of data

All manuscripts must include a data availability statement. This statement should provide the following information, where applicable:

- Accession codes, unique identifiers, or web links for publicly available datasets
- A description of any restrictions on data availability
- For clinical datasets or third party data, please ensure that the statement adheres to our policy

Source data are provided with this paper

Field-specific reporting

Please select the one below that is the best fit for your research. If you are not sure, read the appropriate sections before making your selection.

- [x] Life sciences
- [ ] Behavioural & social sciences
- [ ] Ecological, evolutionary & environmental sciences

For a reference copy of the document with all sections, see nature.com/documents/nr-reporting-summary-flat.pdf

Life sciences study design

All studies must disclose on these points even when the disclosure is negative.

Sample size	No statistical methods was used to predetermine the sample size via calculation. However, a minimal number of 3 samples was used for each sampling point to have statistical power to calculate geometric mean value.
Data exclusions	No data was excluded in the analysis
Replication	The challenge study in mice that were immunized with 1 mcg of inactivated NDV-HXP-S has been replicated 3 times so far (Figure 2, figure 7, unpublished) The immunization study in mice with 10^6 EID50 live NDV-HXP-S has been repeated 4 times (serology, IgA, ICS T cells). The figure 1 SDS-PAGE has been repeated >3 times. Two replicates of SDS-PAGE in Figure Fig.S5 has been performed. The T cell study was performed twice. All other attempts at replication of were successful.
Randomization	Animals were randomized for each group before immunization; When analyzing the data, samples were randomized within each group.
Blinding	Blinding was not applicable to the studies as identification of the animals was required for vaccination

Reporting for specific materials, systems and methods

We require information from authors about some types of materials, experimental systems and methods used in many studies. Here, indicate whether each material, system or method listed is relevant to your study. If you are not sure if a list item applies to your research, read the appropriate section before selecting a response.

Materials & experimental systems

n/a	Involved in the study	
[ ]	[x]	Antibodies
[ ]	[x]	Eukaryotic cell lines
[x]	[ ]	Palaeontology and archaeology
[ ]	[x]	Animals and other organisms
[x]	[ ]	Human research participants
[x]	[ ]	Clinical data
[x]	[ ]	Dual use research of concern

Methods

n/a	Involved in the study	
[x]	[ ]	ChIP-seq
[x]	[ ]	Flow cytometry
[x]	[ ]	MRI-based neuroimaging

Antibodies

Antibodies used	Amersham ECL Mouse IgG HPR-linked whole Ab (from sheep) (Cytiva, NA931, Lot 17246087); HRP-conjugated goat anti-mouse IgG1 (Abcam, ab97240, Lot GR3365481-3); HRP-conjugated goat anti-mouse IgG2a (Abcam, ab97245, Lot GR3362355-3); IgG (H+L) Cross-Adsorbed Goat anti-Hamster, HRP (Invitrogen, HA6007, Lot SC235655); Mouse mAb 1C7C7 from Dr. Thomas Moran (ISMMS, lot 10/22/2020); Goat anti-Mouse IgA Antibody HRP Conjugated (Bethyl laboratories, #90-103P, lot #54); Hamster anti-mouse CD28 (BD Biosciences, 553295, clone 37.51, lot 0357876);Fc Block rat anti-mouse CD16/CD32 (BD Biosciences, 553141, clone 2.4G2, lot 1040760); BV 711 rat anti-mouse CD3 (BioLegend, 100241, clone 17A2, lot B303472); Pacific Blue rat anti-mouse CD4 (BioLegend,100428, clone GK1.5, lot B265546), PerCP/Cy5.5 rat ani-mouse CD8 (BioLegend, 100734, clone 53-6.7, lot B313041); Alexa Fluor 647 rat anti-mouse IFN-γ (BioLegend, 505814, clone XMG1.2, lot B288855), Alexa Fluor 488 rat anti-mouse TNF-α (BioLegend, 506313, clone MP6-XT22, B316861); PE/Cyanine 7 rat-anti mouse IL-2 (BioLegend, 503832, clone JES6-5H4, lot B309408)
Validation	The commercially purchased antibodies were validated by the manufacturers including assays or relevant publications (please refer to the cat. numbers and lot numbers provided above).<br>The mouse monoclonal antibody 1C7C7 has also been commercialized and validated by the manufacturer (Millopore Sigma, ZMS1075) and relevant publications. Other publications include doi: 10.1002/cpmc.108,

Eukaryotic cell lines

Policy information about cell lines

Cell line source(s)	BSRT7 cells were a kind gift from Dr. Benhur Lee at Icahn School of Medicine at Mount Sinai (ISMMS), the cell line was originally developed by Dr. Conzelmann described in DOI: 10.1128/JVI.73.1.251-259.1999. The parent cell line of the BSRT7 is

	BHK-21 clone 13; Vero E6 cells were purchased from ATCC (CRL-1586);Chicken embryo fibroblasts (CEF) were isolated from 10-day old embryonated chicken eggs
Authentication	BSRT7 cells were validated for its stable expression of T7 polymerase by transfecting GFP expression plasmid with a T7 promoter; Vero E6 cells was directly purchased from ATCC and validated by its ability to support SARS-CoV-2 replication; CEF cells were directly isolated by the authors from chicken embryos. No additional authentication was performed.
Mycoplasma contamination	Although cells were not tested for mycoplasma. NDV-HXP-S that was rescued using BSRT7 and CEF cells was tested mycoplasma free by Clongen Laboratories
Commonly misidentified lines (See ICLAC register)	No commonly misidentified lines were used in the study

Animals and other organisms

Policy information about studies involving animals; ARRIVE guidelines recommended for reporting animal research

Laboratory animals	Six to eight-week old female BALB/c mice were purchased from The Jackson Laboratory; Six to eight-week old female Golden Syrian hamsters were purchased from Charles River Laboratories
Wild animals	No wild animals were used in the study
Field-collected samples	The study did not involved in samples collected from the field.
Ethics oversight	All the animal experiments were performed in accordance with protocols approved by the Icahn School of Medicine at Mount Sinai Institutional Animal Care and Use Committee (IACUC)

Note that full information on the approval of the study protocol must also be provided in the manuscript.
